# Biological Foundation Models for Complex Disease Research and Clinical Translation

**DOI:** 10.3390/biology15171527

**Published:** 2026-09-03

**Authors:** Tiana Noll-Walker, Yong Chen

**Affiliations:** Department of Biological and Biomedical Sciences, Rowan University, Glassboro, NJ 08028, USA; nollwa36@rowan.edu

**Keywords:** large language model, biological foundation model, complex disease, single-cell omics, precision medicine

## Abstract

Complex diseases, including cancer, rare genetic disorders, neurodevelopmental and psychiatric conditions, and neurodegenerative diseases, arise from interactions among genetic variation, gene regulation, and cellular states that are difficult to capture using a single data type or biological scale. Biological foundation models address this challenge by treating nucleotides and genes as tokens and learning representations that can be transferred to downstream biomedical and clinical tasks. In this review, we examine two major model classes, genomic sequence foundation models and cell foundation models, and compare their tokenization strategies, model architectures, pretraining objectives, and adaptation methods. We summarize their emerging applications in regulatory variant interpretation, disease-associated cell-state analysis, drug-response prediction, and therapeutic target discovery across complex diseases. We distinguish applications supported by experimental or retrospective validation from those that remain primarily computational or conceptual. We further discuss key challenges to clinical translation, including multimodal data integration, model interpretability, benchmarking, patient-specific prediction, and privacy protection. We highlight future opportunities to integrate biological foundation models with emerging frameworks of medical digital twins, agentic AI, and federated learning. By linking model design to translational goals, this review provides a practical framework for evaluating biological foundation models and their readiness for complex disease research and clinical use.

## Simple Summary

Complex diseases are difficult to study because they involve many interacting genetic and cellular factors. Biological foundation models are new artificial intelligence tools that can learn useful patterns from large biological datasets and apply them to different research questions. This review introduces the major types of these models and explains how they are designed, trained and adapted for biomedical applications. We explain how they may help researchers understand disease mechanisms, identify important genetic changes, predict drug responses, and discover treatment targets. We also discuss current limitations, especially the need for better validation, clearer interpretation, improved data integration, and stronger protection of patient privacy before these models can be widely used in clinical practice.

## 1. Introduction

Complex diseases, including cancer, neurodegenerative disorders, autoimmune conditions, and psychiatric illnesses, arise from interactions among genetic variation, gene regulatory dysregulation, cellular heterogeneity, and environmental exposure [1,2]. Over the past decade, high-throughput sequencing technologies have greatly advanced genomic and biomedical research by generating large-scale datasets across bulk tissue, single-cell, and spatially resolved levels [3,4,5]. These technologies and the resulting datasets provide an unprecedented opportunity to develop biological foundation models that learn generalizable representations from large-scale unlabeled or weakly labeled biological data. Large language model (LLM) approaches offer a powerful framework for modeling biological systems by treating biological entities, such as nucleotides, genes, genomic regions, or cells, as tokens and representing biological information as sequences or structured collections of tokens [6,7,8]. This conceptual shift has led to two major classes of models relevant to complex disease research. The first class, genomic sequence foundation models, is pretrained on large DNA sequence corpora to learn regulatory patterns associated with transcription factor binding, chromatin accessibility, splicing, gene expression, and variant effects [9,10,11]. The second class, cell foundation models, is pretrained on large-scale transcriptomic atlases to learn cell-state representations that can generalize across tissues, disease conditions, perturbations, and patient cohorts [12,13,14].

**Figure 1:**
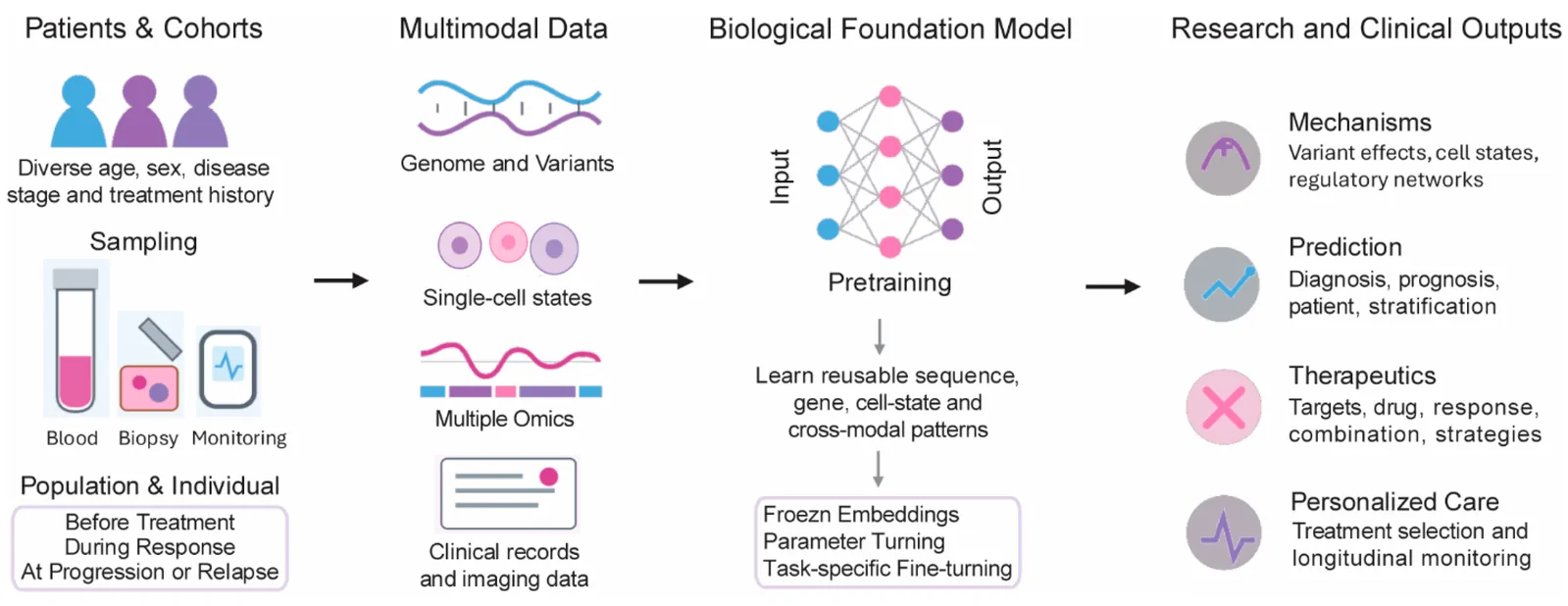
Conceptual framework for applying biological foundation models to complex disease research and precision medicine. Multimodal data from diverse public omics databases and patient cohorts are used to pretrain models that learn shared biological patterns. These models can then be adapted for studying disease mechanisms, predicting clinical outcomes, identifying therapies, and supporting personalized care.

A major strength of biological foundation models is their ability to transfer knowledge from large-scale reference datasets to disease-specific applications with limited labeled data. This capability is especially valuable for complex diseases and clinical settings in which conventional statistical approaches are constrained by small sample sizes, rare cell populations, or highly distributed genetic effects. Rare diseases often have small patient cohorts, limited available tissue samples, and too few labeled variants for reliable statistical analysis or disease-specific model training [15,16,17]. Cancer presents a different challenge because tumors contain mixtures of malignant, stromal, and immune cells, including rare but clinically important populations that may drive metastasis, immune evasion, or treatment resistance. These rare populations can be missed in bulk sequencing because their signals are averaged with those of other cells [18,19]. Psychiatric and neurodegenerative disorders are also difficult to study because they are often influenced by many small-effect noncoding variants [20]. These variants may affect gene regulation in specific cell types, brain regions, or developmental stages, making their functional consequences difficult to interpret without models that connect sequence variation to regulatory and cellular changes [21]. By leveraging representations learned during pretraining from multimodal data, biological foundation models may improve disease mechanism discovery, biomarker identification, variant interpretation, patient stratification, treatment-response prediction, pharmacogenomic analysis, and drug discovery (Figure 1). These representations can then be adapted to investigate disease mechanisms, predict clinical outcomes, identify therapeutic opportunities, and support personalized care. Building on these capabilities, two complementary frameworks may further support the clinical translation of biological foundation models: medical digital twins and agentic AI. Medical digital twins create dynamic computational representations of individual patients by integrating population-level knowledge with patient-specific molecular, cellular, clinical, imaging, and longitudinal data, and can be updated as new information becomes available [22,23]. Agentic artificial intelligence (AI) provides a complementary framework in which autonomous AI agents can coordinate analytical workflows, select and execute appropriate bioinformatics tools, integrate results across data types, and generate task-specific predictions or decision-support outputs [24]. Together, these approaches may help translate foundation-model representations into more individualized, interpretable, and actionable biomedical applications.

In this review, we focus on recent advances in biological foundation models and their applications to complex disease research and clinical translation. We first provide a concise overview of model architectures, tokenization strategies, pretraining objectives, and adaptation methods to establish the technical context needed to understand their downstream capabilities. Rather than providing an exhaustive survey of model engineering, our primary emphasis is on how these models are being applied to biomedical problems, the strength of evidence supporting these applications, and the remaining barriers to clinical translation. We specifically discuss their applications in cancer, rare disease, neurodevelopmental and psychiatric disorders, neurodegenerative disease, personalized treatment, pharmacogenomics, and drug discovery. We highlight key challenges that remain before these models can be broadly translated into clinical research and practice, including the gap between population-level and personalized prediction, model interpretability, data fragmentation, multimodal integration, benchmarking, and clinical validation. The central thesis of this review is that the translational value of biological foundation models depends not simply on model scale or benchmark performance, but on whether model representations and training strategies are aligned with the biological question and supported by appropriate experimental and clinical validation.

## 2. Biological Foundation Models and Architectures

**Figure 2:**
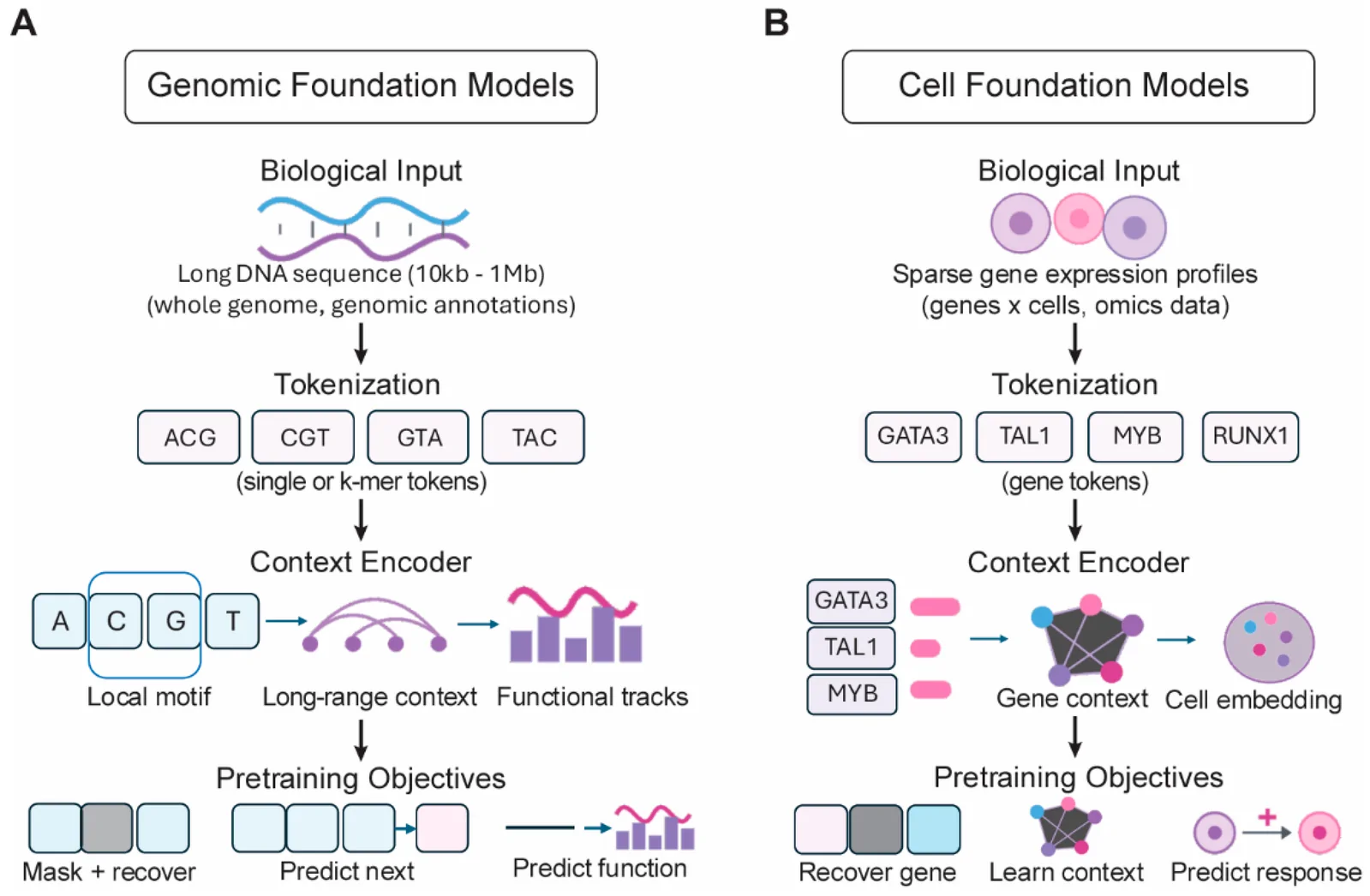
Representative architectures and training strategies of biological foundation models. (**A**) Genomic foundation models operate at the genomic sequence and regulatory-feature level, converting long DNA sequences into tokens and learning local motifs, long-range regulatory context, and functional genomic representations through objectives such as masked-token recovery, next-token prediction, and functional prediction. (**B**) Cell foundation models operate at the gene-expression and cellular-state level, learning gene–context relationships, cell embeddings, and cellular programs from sparse single-cell expression profiles through objectives such as masked-gene recovery, context learning, and perturbation-response prediction.

Biological foundation models used in complex disease research can be broadly grouped into two complementary classes: genomic foundation models and cell foundation models. Genomic foundation models learn from DNA sequence and are designed to capture regulatory grammar, including sequence motifs, chromatin-associated signals, splicing elements, gene-expression regulation, and variant effects. Cell foundation models learn from multi-omic profiles and are designed to capture cell identity, cell-state transitions, perturbation responses, and disease-associated cellular heterogeneity. These two model classes operate at different biological scales: genomic models connect sequence variation to molecular regulation, whereas cell models connect gene-expression patterns to cellular phenotype and tissue-level disease mechanisms. As illustrated in Figure 2, genomic foundation models encode long DNA sequences to learn local motifs, long-range interactions, and functional regulatory patterns, whereas cell foundation models encode sparse gene-expression profiles to learn gene–context and cell-level representations. These models are pretrained using objectives such as masked-token recovery, next-token prediction, gene-expression reconstruction, and cellular-response prediction.

The core architectural consideration in genomic modeling is how to represent long, information-dense DNA sequences efficiently while preserving biologically meaningful context. Early transformer-based models, such as DNABERT [9], DNABERT-2 [25], and the Nucleotide Transformer [10], used nucleotide or k-mer tokenization with masked-token prediction to learn local regulatory patterns. These models are useful for promoter prediction, transcription-factor binding analysis, splice-site detection, and variant-effect scoring. However, many disease-relevant regulatory mechanisms depend on distal enhancer–promoter interactions and other long-range regulatory relationships that cannot be adequately captured by short sequence windows [26,27]. To address this limitation, newer models have adopted long-context or hybrid architectures. For example, Enformer [28] and Borzoi [29] combine convolutional or U-Net-like components with attention-based modeling to improve sequence-to-function prediction over longer genomic regions, while AlphaGenome extends this direction by predicting multiple regulatory outputs from genomic sequences [11]. Evo 2 uses a sub-quadratic sequence modeling architecture to support genome-scale context and generative sequence modeling [30]. These models reflect a major transition in genomic foundation modeling, shifting from local motif recognition toward long-range, multimodal, and function-oriented regulatory prediction.

Despite these advances, longer context length alone does not solve all challenges in disease modeling. Many genomic models are trained primarily on reference genomes or population-level regulatory datasets, which may limit their ability to predict personalized effects of combinations of variants, rare noncoding mutations, or disease-specific regulatory states [31,32,33]. DNA sequence provides regulatory potential, but actual gene regulation depends on cell type, transcription-factor availability, chromatin state, environmental cues, and disease context. Therefore, multimodal models that integrate sequence with chromatin accessibility, histone modifications, gene expression, or three-dimensional chromatin structure are increasingly important. Models such as AlphaGenome [11] and ChromoGen [34] demonstrate how incorporating regulatory or epigenomic context can improve biological interpretation, but their performance remains constrained by the diversity, resolution, and disease relevance of the training data.

Cell foundation models face a different representational problem. Instead of modeling a linear nucleotide sequence, they must convert a sparse, high-dimensional gene-expression vector into a format suitable for neural network learning. Current models use several strategies. Geneformer represents each cell as a rank-ordered sequence of genes, emphasizing relative expression within a cell [35]. scGPT represents genes together with discretized expression values, allowing the model to learn both gene identity and expression magnitude [12]. scFoundation uses continuous expression representations and an asymmetric encoder–decoder design to reconstruct full transcriptomic profiles from sparse single-cell inputs [13]. UCE further shifts the emphasis from task-specific prediction to universal cell representation by incorporating protein-language-model-derived gene embeddings and cross-species information [14]. Other models, such as CellFM [36], scPRINT [37], and RegFormer [38], introduce scalable architectures to improve cell-state modeling, gene-network inference, and perturbation prediction. Spatial transcriptomic foundation models represent a related extension of the architectures reviewed here by incorporating tissue spatial context along with gene-expression information. ST-Align, the first multimodal foundation model for spatial transcriptomics, was pretrained on ~1 million paired spot-and-niche-level image-gene samples from ~600 human tissue samples that included normal, diseased and cancerous states. On human dorsolateral prefrontal cortex tissue, it improved spatial domain identification by ~8% over unimodal transcriptomic FMs and existing multimodal frameworks [39]. STORM, which was pretrained on spatially resolved spots with matched histology across 18 organs, directly predicts spatial gene expression from routine tissue images. In kidney cancer, it identified tumor subdomains with distinct metabolic and inflammatory signatures whose marker gene expression predicted shorter progression-free survival in an independent cohort, and its external validation cohorts included glioblastoma [40]. Together, these applications suggest that spatial context represents an important next dimension for extending genomic sequence and cell foundation models.

The choice of tokenization and input representation is especially important for disease applications. Rank-based gene representations reduce sensitivity to sequencing depth but may discard absolute expression differences that are biologically meaningful. Discretized expression bins preserve coarse magnitude information but impose artificial boundaries on continuous expression values. Continuous representations retain more quantitative information but require architectures that can handle sparsity, dropout, and the large gene vocabulary of single-cell data. Similarly, genomic tokenization strategies involve trade-offs between nucleotide-level precision, k-mer-based motif representation, and learned compression. These design choices determine which biological signals are visible to the model and which may be lost before training begins.

For downstream biomedical and clinical tasks, pretrained models are typically used in three ways: frozen feature extraction, full fine-tuning, or parameter-efficient fine-tuning. Frozen embeddings are computationally efficient and can be effective when the pretrained representation is well aligned with the downstream task. Full fine-tuning can improve task-specific performance but requires more labeled data and computational resources and may overwrite general biological representations [41]. Parameter-efficient strategies, such as adapters or low-rank adaptation (LoRA) [42], offer a practical compromise by updating only a small subset of parameters while preserving the pretrained backbone. This is particularly relevant for complex disease studies, where patient-level datasets are often small, heterogeneous, and expensive to generate.

In practice, biological LLM architectures should be selected according to the biological question and data scale. Genomic sequence models are most appropriate for variant interpretation, regulatory-element prediction, splicing analysis, and sequence design. Cell foundation models are better suited for cell-type annotation, disease-state classification, perturbation-response prediction, cell-state modeling, and gene-network inference. Multimodal and cross-scale models will likely be required to connect genetic variation to cell-type-specific regulatory changes, altered cellular states, and disease phenotypes. Thus, the central architectural consideration is not simply model size, but whether the model representation, context length, learning objective, and adaptation strategy match the biological mechanism being studied.

## 3. Biomedical Applications and Clinical Relevance

Having established the major model classes and the architectural features that shape their capabilities, we now focus on their biomedical applications and the evidence supporting their translational potential. Representative applications of genomic and cell foundation models across complex disease research are summarized in Table 1. Across these studies, most current applications demonstrate computational performance, retrospective association, or mechanistic plausibility, whereas prospective patient-level validation remains limited. Several models have recapitulated experimentally established regulatory mechanisms or shown agreement with independent biological measurements, while others have primarily demonstrated improved prediction or representation learning in research datasets. Therefore, a key distinction among current applications is not simply what tasks foundation models can perform, but how rigorously their predictions have been validated and how close they are to clinical use. This evidence gap is particularly important for applications involving treatment selection, prognosis, and individualized prediction, where retrospective benchmark performance alone is insufficient to establish clinical utility.

### 3.1. Cancer Studies

Cancer is one of the most immediate application areas for biological foundation models because tumors contain extensive genetic, transcriptional, and cellular heterogeneity. Cell foundation models are well suited to this challenge because they learn cell-state representations from large transcriptomic atlases and can then be adapted to identify disease-associated cell populations in patient samples [43,44]. Recent studies illustrate this potential. For example, scGPT has been applied to acute lymphoblastic leukemia samples to distinguish rare cell subtypes and identify transcriptional pathways associated with immune evasion and abnormal proliferation [12,45]. CellFM has been used to distinguish activated immune cell states in cancer contexts, which is relevant to immunotherapy response prediction [36]. These applications suggest that foundation model embeddings may improve the resolution of tumor microenvironment analysis beyond conventional clustering or marker-based annotation.

Cell foundation models also have potential value in drug-response prediction. scFoundation-derived embeddings have been used to represent tumor transcriptomic states for predicting drug sensitivity, improving the prediction of half-maximal inhibitory concentration, a commonly used measure of drug sensitivity, across multiple drugs and cancer types [13]. If validated prospectively, such approaches could support transcriptome-informed treatment selection by ranking candidate therapies before treatment initiation. However, clinical use will require validation in independent patient cohorts, comparison with existing molecular signatures, and careful assessment of robustness across sequencing platforms and cancer subtypes.

**Figure 3:**
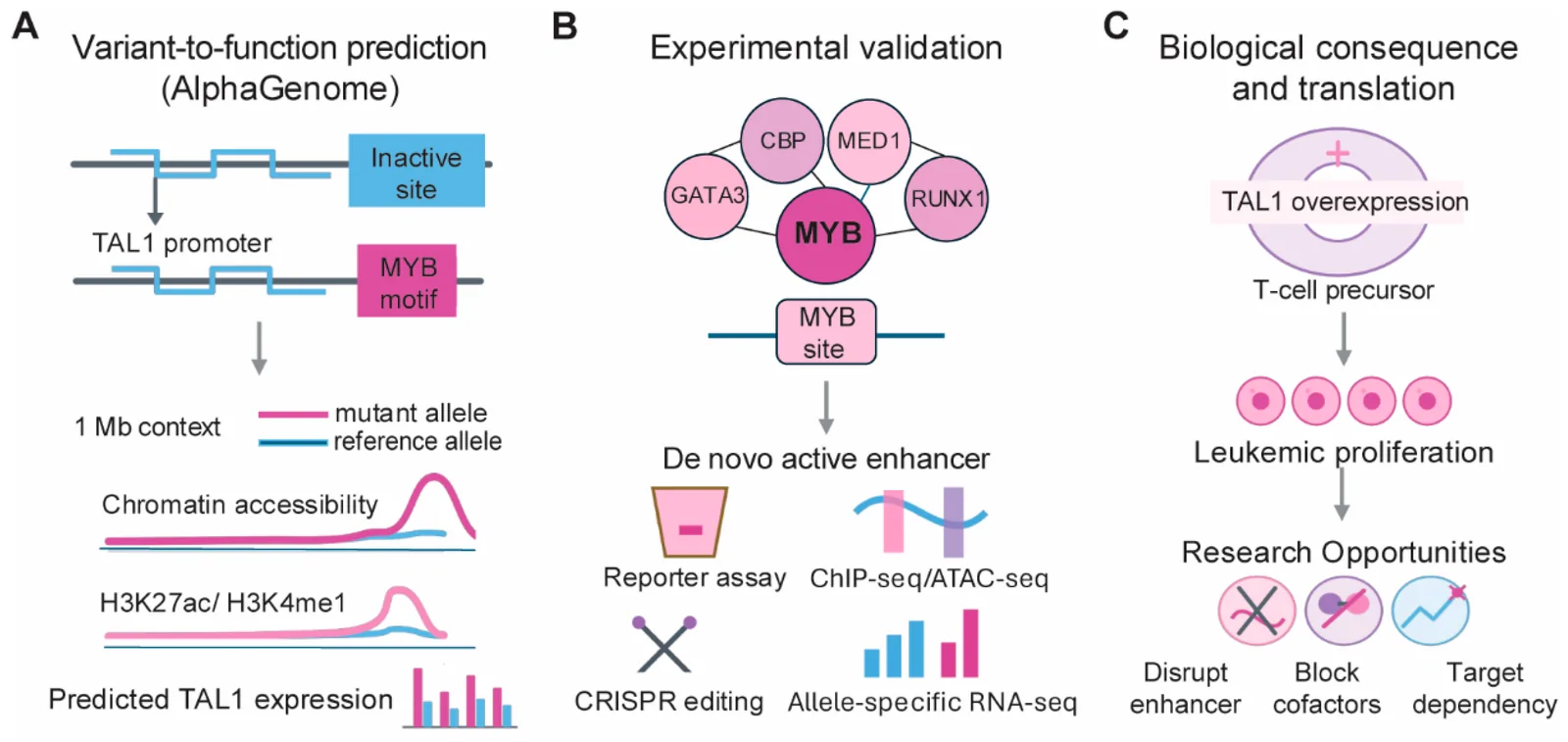
Validation and interpretation of AlphaGenome-predicted regulatory variants. (**A**) AlphaGenome predicts how a noncoding variant near the TAL1 locus alters chromatin accessibility, histone modifications, and gene expression. (**B**) The predicted enhancer activity is evaluated using reporter assays, chromatin profiling, clustered regularly interspaced short palindromic repeats (CRISPR) editing, and allele-specific RNA sequencing. (**C**) Increased TAL1 expression promotes abnormal T-cell proliferation and provides opportunities to study disease mechanisms and therapeutic targets.

Genomic foundation models provide a complementary route for cancer variant interpretation. Many cancer driver events occur in noncoding regions and remain difficult to classify using standard variant annotation pipelines. Models such as AlphaGenome can predict the effects of noncoding variants across regulatory outputs, including chromatin accessibility, transcription factor binding, splicing, and gene expression [11]. A representative example involved a known oncogenic insertion near TAL1 in T-cell acute lymphoblastic leukemia. AlphaGenome predicted the formation of a MYB-associated regulatory element, increased chromatin accessibility and activating histone marks, and increased TAL1 expression ~7.5 kb from the insertion, thereby recapitulating the previously established regulatory mechanism (Figure 3). This example demonstrates how a foundation model can connect variant-effect prediction with experimental validation through reporter assays, chromatin profiling, CRISPR editing, and allele-specific RNA sequencing, thereby linking computational variant interpretation to disease mechanisms and potential therapeutic targets.

### 3.2. Rare Disease

Rare diseases present a major data-scarcity challenge. Patient cohorts are often too small for conventional statistical modeling, disease-relevant tissues may be difficult to obtain, and many candidate variants lack functional annotation. Foundation models are attractive in this setting because they learn general biological representations from large public datasets during pretraining and can then be adapted to small disease-specific datasets [46,47]. Geneformer provides an example of this strategy (Table 1). After pretraining on millions of single-cell transcriptomes, it was fine-tuned on a limited number of cardiomyopathy patient samples and used for in silico perturbation analysis to prioritize candidate network regulators and potential therapeutic targets [35]. Similarly, cell foundation models could be adapted to other rare disorders with limited patient cohorts, including neurological, developmental, and autoimmune diseases for which disease-relevant cellular models are available.

At the sequence level, evolutionary and genomic foundation models can support rare variant interpretation. For example, GPN-Star uses evolutionary constraint learned from multiple aligned genomes to predict the functional effects of variants in both coding and noncoding regions [48]. Similarly, AlphaGenome predicts the regulatory consequences of noncoding variants by modeling their effects on chromatin accessibility, transcription factor binding, histone modifications, splicing, and gene expression [11]. These models can help prioritize candidate disease-causing variants, particularly in patients harboring multiple variants of uncertain significance. However, successful clinical translation will require integrating variant predictions with inheritance patterns, phenotype matching, functional genomics, and expert clinical interpretation rather than relying on computational predictions alone.

### 3.3. Neurodevelopmental, Psychiatric, and Neurodegenerative Disorders

Neurological and psychiatric disorders are especially challenging because disease risk is often distributed across many noncoding variants, cell types, developmental stages, and brain regions. Genomic foundation models may help prioritize regulatory variants from genome-wide association studies by identifying variants more likely to affect brain-specific regulatory programs [28]. This is particularly relevant for disorders such as autism spectrum disorder, schizophrenia, and Alzheimer’s disease, in which many risk loci lie outside protein-coding regions and require functional interpretation. More broadly, previous studies and reviews of AI and LLM applications in neurological and psychiatric disorders highlight the need for improved computational approaches to connect complex molecular variation with disease-relevant biological mechanisms [49,50]. However, disease-specific genomic foundation models tailored to neurodevelopmental, psychiatric, and neurodegenerative disorders remain limited, highlighting an important opportunity for future model development using brain-specific regulatory, developmental, and disease-associated genomic data.

Cell foundation models provide a complementary approach by analyzing disease-associated cell states. As single-cell and single-nucleus atlases of the human brain continue to expand, models such as scGPT, Geneformer, and CellFM could be applied to patient-derived neurons, glia, brain organoids, or postmortem tissue to identify disrupted transcriptional programs and vulnerable cell populations. For neurodegenerative diseases, these models may be useful for detecting gradual transitions from healthy to disease-associated cell states and for prioritizing perturbations that could reverse or stabilize pathological transcriptional programs [51,52]. For example, scGPT was fine-tuned on a multiple sclerosis dataset and classified disease states from single-cell profiles with ~85% accuracy. However, clinical translation in neurological disease remains particularly difficult. Brain tissue is often inaccessible, postmortem samples may reflect late-stage disease, and in vitro models may not fully reproduce human brain development or degeneration [53]. Therefore, foundation model predictions in these areas should be viewed primarily as hypothesis-generating until supported by experimental or longitudinal clinical validation.

### 3.4. Personalized Treatment and Pharmacogenomics

Personalized medicine requires predicting how a patient’s molecular profile influences disease progression and treatment response. Foundation models may contribute to this goal by integrating genomic variation, cell-state information, and perturbation-response patterns [54]. Cell models can represent patient-specific transcriptional states and predict how cells may respond to genetic or pharmacological perturbations. Genomic models can prioritize regulatory variants that affect drug targets, immune pathways, or tissue-specific expression programs. In general, genomic and cell foundation models can further support personalized medicine by integrating patient-specific molecular, clinical, and longitudinal data to improve disease characterization, treatment-response prediction, and clinical decision support.

In oncology, transcriptomic embeddings generated by models such as scFoundation can be used as low-dimensional representations of tumor cell states for downstream prediction of drug sensitivity, treatment response, and patient stratification. In one representative retrospective analysis, scFoundation-derived embeddings increased the correlation between predicted and measured responses to a CDK inhibitor from 0.07 to 0.73, illustrating how pretrained transcriptomic representations may improve drug-response prediction while still requiring prospective patient-level validation (Table 1). These models are typically pretrained on large-scale scRNA-seq datasets using self-supervised objectives that capture gene–gene dependencies, co-expression patterns, and cell-state variation. The resulting embeddings can then be transferred to cancer-specific datasets and combined with drug-response labels, genomic alterations, pathway activity scores, or clinical covariates to train predictive models. Perturbation-oriented models such as Geneformer and CellFM may further support target discovery by estimating how changes in the expression or activity of specific genes alter a cell’s position in the learned representation space. In principle, in silico gene knockout, overexpression, or ranking-based perturbation analyses can be used to prioritize interventions predicted to move malignant cells away from proliferative, drug-resistant, or immune-evasive states and toward healthier or treatment-sensitive phenotypes. These approaches have shown promise in cancer-focused case studies, but their clinical translation will require standardized preprocessing and gene-mapping procedures, careful control of batch and tissue effects, and evaluation across diverse tumor types, treatment regimens, and patient populations. While cancer currently provides the most published evidence for foundation-model-driven treatment-response prediction, the underlying strategies are not cancer-specific. Geneformer’s in silico perturbation approach has similarly been used to prioritize candidate therapeutic targets in cardiomyopathy, and scGPT-based disease-state classification has been demonstrated in multiple sclerosis.

Genomic foundation models may contribute to pharmacogenomic research by improving the functional interpretation of noncoding variants, but this represents an upstream step rather than direct clinical pharmacogenomic implementation. By comparing model predictions for reference and alternative alleles, these models can estimate potential effects on transcription factor binding, chromatin accessibility, histone modifications, enhancer and promoter activity, splicing, and tissue-specific gene expression [55,56]. These allele-specific scores could be integrated with expression quantitative trait loci, epigenomic annotations, and gene regulatory networks to determine whether a patient’s variants affect genes involved in drug metabolism, transport, pharmacodynamic response, or toxicity. Clinical interpretation would require conversion of variants into haplotypes, diplotypes, and predicted metabolizer phenotypes, followed by integration with PharmGKB evidence and CPIC prescribing recommendations [57,58,59]. Translation into clinical practice will also require experimental calibration, incorporation of dosage, drug–drug interactions, organ function, environmental exposures, and integration with electronic health records and clinical decision-support systems [60,61]. Thus, foundation-model predictions should currently be viewed as complementary evidence for variant interpretation rather than as stand-alone tools for clinical treatment selection.

### 3.5. Drug Discovery and Target Identification

Biological foundation models may also accelerate early-stage drug discovery by prioritizing targets, perturbations, and molecular mechanisms before experimental screening. Cell foundation models can simulate or predict transcriptional responses to gene perturbations, enabling computational screening of candidate targets across disease-relevant cell types [52,62,63]. This could reduce the number of hypotheses requiring expensive experimental validation. Geneformer’s in silico perturbation framework illustrates this direction by ranking genes according to their predicted ability to shift disease-associated cell states toward healthier profiles [35]. CellFM and related models further extend perturbation analysis by learning relationships among genes, cell states, and transcriptional responses at large scale. These approaches are particularly useful for diseases in which target discovery depends on identifying cell-type-specific regulatory programs rather than single-gene associations.

Generative genomic models such as Evo [64] and Evo 2 [30] suggest another future direction: the design of functional biological sequences. Although current demonstrations are strongest in microbial or synthetic biology settings, similar principles may eventually support the design of regulatory elements, promoters, enhancers, or gene therapy vectors with desired tissue-specific activity. Such applications will require stringent experimental validation and safety evaluation before biomedical translation. The biomedical relevance of biological foundation models lies not in replacing experimental or clinical decision-making, but in improving prioritization, interpretation, and hypothesis generation. Their most immediate value is likely to be in variant interpretation, disease-cell-state discovery, drug-response prediction, and target prioritization. Their broader clinical impact will depend on prospective validation, mechanistic interpretability, reproducibility across datasets and platforms, and responsible integration into existing biomedical workflows.

## 4. Open Problems and Future Directions

A major challenge for the clinical translation of biological foundation models is bridging the gap between population-level disease modeling and patient-specific diagnosis and treatment. Population-level models can identify reproducible pathways, biomarkers, disease subtypes, and treatment-response patterns, but these averaged signals may not accurately predict how an individual patient will progress or respond to a specific therapy. Medical digital twins provide a potential framework for addressing this challenge by integrating mechanistic models learned from reference populations with patient-specific molecular, clinical, imaging, and longitudinal data [22,23]. As new measurements become available, the digital twin can be updated to simulate disease trajectories, evaluate alternative interventions, and support individualized decision-making. However, the accuracy of such predictions remains limited by biological heterogeneity, incomplete mechanistic knowledge, sparse longitudinal data, and uncertainty in patient-specific measurements [65]. Biological foundation models could strengthen digital twin frameworks by integrating multi-omics data and transferring representations learned from large populations to individual patients [66]. Nevertheless, current models remain difficult to interpret and may learn statistical correlations rather than causal mechanisms, limiting their ability to explain disease progression or reliably forecast treatment outcomes.

One promising direction is to build agentic AI platforms that combine biological foundation models with validated bioinformatics tools, rather than relying on pretrained biological LLMs alone [67]. In such a framework, foundation models could provide representation learning, biological reasoning, and hypothesis generation, while AI agents would plan and execute complete analytical workflows by selecting appropriate high-performance tools for specific tasks [24]. Technically, an agentic platform could use established pipelines for read alignment, variant calling, single-cell preprocessing, batch correction, differential expression analysis, pathway enrichment, cell–cell communication inference, perturbation analysis, and spatial transcriptomics integration. The agent would not replace these specialized tools, but would coordinate them, check input formats, monitor quality-control metrics, compare alternative methods, summarize results, and maintain reproducible records of parameters, software versions, and intermediate outputs. Such platforms could also integrate patient-specific data with public reference atlases and foundation-model embeddings to support digital twin construction. Importantly, this tool-using agentic strategy may improve reliability and interpretability by grounding AI-generated hypotheses in transparent, benchmarked computational analyses rather than in model predictions alone.

Evidence for the clinical effectiveness of digital twins and foundation-model-enabled precision medicine remains limited. However, early studies suggest potential benefits for personalized health management, treatment selection, and individual risk prediction. A recent review of digital twins in precision health identified only 12 eligible studies and reported favorable results for 36 of 45 measured outcomes, including personalized health management, treatment selection, and individual risk prediction [68]. However, only one study was a randomized controlled trial; many studies relied on virtual rather than real patients, lacked appropriate control groups, or did not include follow-up measurements. The review also found limited multicenter implementation and emphasized the need for high-quality clinical trials, real patient feedback, and evaluation across diverse populations and healthcare systems. These findings suggest that although digital twins and biological foundation models are promising, their clinical value must be demonstrated through prospective validation, transparent uncertainty estimation, and careful integration into existing clinical workflows.

Another major barrier is data fragmentation. Clinically relevant genomic, single-cell, imaging, and electronic health record data are distributed across hospitals, research centers, and biobanks, often under strict privacy, consent, and regulatory constraints. Because biological foundation models require large and diverse datasets for robust training and validation, fragmented data access can reduce model generalizability, reproducibility, and fairness across independent patient populations. Federated learning offers one potential solution by allowing multiple institutions to collaboratively train shared models while keeping sensitive patient data local [69,70]. Instead of transferring raw genomic or clinical data to a central repository, participating sites exchange model updates or parameters, thereby reducing privacy risks and supporting compliance with regulations. However, federated learning does not eliminate all privacy and governance concerns. Model updates may still leak sensitive information through inference or reconstruction attacks, and increasingly rich multimodal patient representations may further exacerbate these privacy risks. Simulated attacks in synthetic genomic federated learning settings have shown that gradient-based membership inference can identify training-set membership with a precision of 0.79 and an F1 score of 0.87, suggesting that exchanging model updates alone does not necessarily guarantee privacy protection, even before considering the additional complexity of real-world data [71]. Therefore, future clinical deployment of biological foundation models will require not only federated learning, but also stronger privacy-preserving methods such as secure aggregation, differential privacy, encryption-based computation, and transparent data-governance frameworks. Addressing these challenges will be essential for developing foundation models that are clinically useful, reproducible, privacy-preserving, and equitable across diverse patient populations.

## 5. Conclusions

Biological foundation models are rapidly emerging as powerful tools for complex disease research by enabling transferable representation learning across genomic, transcriptomic, and multimodal biological data. Rather than attempting an exhaustive comparison of foundation-model architectures, this review has focused on how key model-design choices translate into biomedical capabilities and, importantly, on the level of evidence supporting their progression toward clinical application. Genomic sequence foundation models provide new opportunities for regulatory variant interpretation and sequence-to-function prediction, while cell foundation models support disease-state characterization, perturbation analysis, drug-response prediction, and therapeutic target discovery. Although these approaches have shown substantial promise across cancer, rare diseases, neurological disorders, and personalized medicine applications, most applications remain at the computational or early experimental stage and require further prospective and clinical validation. Future progress will depend on improving model interpretability, multimodal integration, reproducibility, patient-specific prediction, and data privacy. Emerging frameworks such as medical digital twins, agentic AI, and privacy-preserving federated learning may further extend the utility of biological foundation models by connecting large-scale biological representations with individualized analysis and clinical decision support. Ultimately, the successful translation of these models will require close integration of computational innovation with experimental validation, clinical expertise, and responsible data governance.

## Figures and Tables

**Table 1:** Representative biomedical applications and translational evidence for biological foundation models. The models are grouped by class, with the first five representing genomic foundation models and the remaining six representing cell foundation models.

Biomedical Question	Biological Context	Model	Representative Published Evidence	Potential Biomedical Use	Validation Status	PMID/Links
Can a noncoding cancer variant be linked to its regulatory mechanism?	T-cell acute lymphoblastic leukemia (T-ALL); TAL1; CD34+ common myeloid progenitor tracks	AlphaGenome	AlphaGenome evaluated a known oncogenic insertion near TAL1 and predicted the creation of a MYB-associated regulatory element, increased chromatin accessibility and activating histone marks, and increased TAL1 expression ~7.5 kb away. The predictions recapitulated the previously established mechanism.	Prioritize noncoding variants and nominate affected genes, cell types, and experimental validation assays.	Retrospective mechanistic validation; no prospective diagnostic evaluation.	41606153
Can DNA sequence predict tissue-specific transcriptional and splicing effects of variants?	GTEx human tissues; example loci include INSR in adipose tissue	Borzoi	Borzoi predicted tissue-specific RNA-seq coverage from 524-kb DNA sequences and scored variant effects on transcription, splicing, and polyadenylation. It outperformed or matched models trained for narrower regulatory tasks in multiple quantitative-trait-locus evaluations.	Prioritize variants that may alter gene expression or splicing and select tissue-specific assays for follow-up.	Retrospective genomic and eQTL benchmarking; limited evidence for individualized prediction.	39779956
Can long-range regulatory effects be inferred directly from DNA sequence?	Multiple human tissues and loci, including CD44 and LDLR regulatory regions	Enformer	Enformer incorporated sequence information from up to ~100 kb away and improved prediction of gene expression, enhancer-promoter interactions, and experimentally measured variant effects relative to earlier sequence models. However, performance across genetically distinct individuals was substantially weaker than across-gene benchmark performance.	Fine-map disease-associated noncoding regions and nominate enhancers, promoters, or variants for functional testing.	Experimentally benchmarked regulatory prediction; not reliable as a stand-alone patient-specific predictor.	34608324
Can cell-type-specific three-dimensional chromatin organization be estimated when direct structural measurements are unavailable?	GM12878 lymphoblastoid cells and IMR-90 lung fibroblasts	ChromoGen	ChromoGen generated single-cell three-dimensional chromatin conformations from DNA sequence and DNase-seq data. Predicted structures reproduced experimental TAD boundaries, looping features, and population-level Hi-C patterns and transferred to a cell type excluded from training.	Generate hypotheses about altered enhancer-promoter contacts or chromatin domains and prioritize regions for Hi-C, imaging, or perturbation studies.	Experimental agreement in cell-line datasets; disease-specific and clinical validation remains to be established.	39888999
Can coding and noncoding variants be prioritized when functional data or patient cohorts are limited?	Rare genetic disease, complex traits, and schizophrenia; genome-wide and brain-specific analyses	GPN-Star	Phylogeny-informed models trained across vertebrate, mammalian, and primate evolutionary timescales improved pathogenic-variant classification, GWAS fine-mapping, heritability enrichment, and rare-variant association testing. The primate model was particularly informative for highly polygenic traits such as schizophrenia, and brain-specific scores showed the strongest schizophrenia enrichment.	Rank variants from whole-genome sequencing, prioritize noncoding variants for functional testing, and improve gene discovery in small rare-disease cohorts.	Large retrospective benchmark and biobank analysis; preprint; no prospective diagnostic validation.	41000926
Which regulatory genes might reverse a disease-associated cell state when patient data are limited?	Cardiomyopathy; cardiac cells and disease-associated gene networks	Geneformer	Geneformer was pretrained on ~30 million single-cell transcriptomes and subsequently fine-tuned on disease-specific data for in silico perturbation analysis. It prioritized candidate network regulators and potential therapeutic targets associated with cardiomyopathy.	Rank candidate genes for functional experiments or early-stage therapeutic target discovery in diseases with small cohorts.	Disease-focused computational validation; experimental and clinical validation of candidate targets is still required.	37258680
Can a pretrained model annotate unfamiliar or heterogeneous single-cell samples?	Single-cell datasets, including immune and multiple-sclerosis-related data	scGPT	scGPT was pretrained on more than 33 million cells and adapted for cell-type annotation, dataset integration, perturbation-response prediction, and gene-network inference. It demonstrated that a shared pretrained representation could be transferred to new single-cell datasets.	Identify cell populations, compare diseased and control samples, and generate hypotheses about altered cell states or gene programs.	Validated on research datasets; not established for clinical classification.	38409223
Can transcriptomic profiles help predict drug sensitivity?	Cancer cell lines and tumor transcriptomes across multiple tissues and drugs	scFoundation	scFoundation embeddings improved tissue-level and single-cell drug-response prediction. In one example, the correlation between predicted and measured responses to a CDK inhibitor increased from 0.07 to 0.73.	Rank compounds for laboratory testing or stratify samples in retrospective drug-response studies.	Retrospective benchmarking and prediction; prospective patient-level utility has not been demonstrated.	38844628
Can a model distinguish clinically relevant immune or tumor cell states and predict perturbation responses?	Human immune cells and tumor single-cell datasets, including basal cell carcinoma and liver cancer	CellFM	CellFM, pretrained on 100 million human cells, outperformed comparison models in cell annotation, perturbation-response prediction, gene-function prediction, and gene–gene relationship inference. It also demonstrated reverse perturbation prediction from observed cell states.	Characterize tumor heterogeneity, distinguish immune-cell states, and prioritize genes that may drive an observed transcriptional phenotype.	Research benchmarking across collected datasets; no prospective treatment-selection validation.	40393991
Can spatial context improve tissue domain identification and gene expression prediction from paired histology and transcriptomic data?	Human brain tissue; 573 tissue slides across normal, diseased and cancerous states	ST-Align	ST-Align was pretrained on 1.3 million spot- and niche-level image-gene pairs, improved spatial domain identification by 7.7% over unimodal transcriptomic foundation models and by 8–9% over existing multimodal frameworks (adjusted Rand index) on human brain tissue.	Identify tissue architecture and gene expression patterns from paired histology and transcriptomic data, reducing reliance on costly single-spot sequencing	Retrospective benchmarking on research datasets; no clinical or prospective validation	arXiv:2411.16793
Can spatial gene expression and tumor microenvironment structure be inferred directly from routine histology images?	Kidney, lung, colorectal, and other tumor types across 18 organs; glioblastoma included in external validation cohorts	STORM	STORM, pretrained on 1.2 million spatially resolved spots with matched histology, predicted spatial gene expression from H&E images and identified tumor subdomains in kidney cancer whose marker gene expression predicted shorter progression-free survival in an independent cohort (TCGA-KIRC).	Infer spatial gene expression and tumor microenvironment structure from routine H&E slides, and stratify patients by prognosis without requiring costly spatial transcriptomics assays.	Retrospective benchmarking and cohort-based prognosis prediction; no prospective clinical validation	arXiv:2604.03630

Note: Representative examples are organized by the biomedical question addressed, biological context, published finding, potential use, and current validation status. Validation status reflects the evidence reported in the cited study and should not be interpreted as clinical approval or readiness. Abbreviations: CDK, cyclin-dependent kinase; eQTL, expression quantitative trait locus; GWAS, genome-wide association study; T-ALL, T-cell acute lymphoblastic leukemia; TAD, topologically associating domain.

## Data Availability

No new data were created or analyzed in this study. Data sharing is not applicable to this article.
